# Optimization and Validation of Dispersive Liquid–Liquid Microextraction for Simultaneous Determination of Aflatoxins B1, B2, G1, and G2 in Senna Leaves and Pods Using HPLC-FLD with Pre-Column Derivatization

**DOI:** 10.3390/toxins15040277

**Published:** 2023-04-07

**Authors:** Thanapoom Maneeboon, Chananya Chuaysrinule, Warapa Mahakarnchanakul

**Affiliations:** 1 Scientific Equipment and Research Division, Kasetsart University Research and Development Institute (KURDI), Kasetsart University, Bangkok 10900, Thailand; rdicnc@ku.ac.th; 2 Department of Food Science and Technology, Faculty of Agro-Industry, Kasetsart University, Bangkok 10900, Thailand; fagiwpm@ku.ac.th

**Keywords:** DLLME, mycotoxin, aflatoxins, HPLC-FLD, medicinal herb, senna

## Abstract

Dispersive liquid–liquid microextraction (DLLME) was optimized for the simultaneous extraction of aflatoxins (AFB1, AFB2, AFG1, and AFG2) from powdered senna leaves and pods. Detection was performed using high-performance liquid chromatography with fluorescence detection (HPLC-FLD) and pre-column derivatization. The parameters affecting the DLLME extraction efficiency were evaluated. Chloroform (200 µL) was used as an extraction solvent, 500 µL of distilled water was used as a dispersive solvent, and the extraction was performed at pH 5.6 with no salt added. The optimized method was validated using leaves and pods according to the European Commission guidelines. The linear range for all aflatoxins was 2–50 µg/kg, with values for regression coefficients of determination exceeding 0.995. The recoveries of spiked senna leaves and pods were in the ranges of 91.77–108.71% and 83.50–102.73%, respectively. The RSD values for intra-day and inter-day precisions were in the ranges of 2.30–7.93% and 3.13–10.59%, respectively. The limits of detection and quantification varied in the ranges of 0.70–1.27 µg/kg and 2.13–3.84 µg/kg, respectively. The validated method was successfully applied for the quantification of aflatoxins in 60 real samples of dried senna leaves and pods.

## 1. Introduction

Senna (*Senna alexandrina* or *Cassia angustifolia*; Thai name: ‘ma kham khaek’) is a traditional herbal medicine that belongs to the *Fabaceae* family. This plant is widespread in tropical regions of East Africa and Asia. Senna is an important medicinal herb used worldwide for treating constipation due to its laxative property [1]. It is also used as a skin disease medication and blood purifier [2]. The dried leaves and pods are the most common plant parts used for medicinal properties [3].

Traditionally, medicinal herbs are cultivated mainly in tropical and subtropical regions which are also favorable for fungal growth and mycotoxin production [4]. Improper agricultural and harvesting practices and poor storage conditions have major implications on the quality and safety of medicinal herbs [5]. For example, inadequate drying or slow drying with low heat over long periods enables the contamination of these products with mycotoxins [6].

Aflatoxins B1 (AFB1), B2 (AFB2), G1 (AFG1), and G2 (AFG2) are regarded as being carcinogenic, mutagenic, and hepatogenic, and very harmful to humans [7]. Of these, AFB1 is considered the most toxic aflatoxin and the most frequent metabolite that occurs in contaminated foodstuffs [8]. The International Agency for Research on Cancer (IARC) classified the AFB1 and naturally occurring mixture of aflatoxins as carcinogenic to humans (Group 1) [9]. *Aspergillus flavus* and *A. parasiticus* are the major producers of aflatoxins, and are responsible for the largest proportion of these mycotoxins occurring in various agricultural commodities [10]. 

The incidence of aflatoxins in medicinal herb products has been reported in several countries [11,12,13]. Recently, the mean level of AFB1 and total aflatoxins in spice and herbal samples collected from Polish retail markets were found with concentration ranges of 0.6–4.5 µg/kg and 0.3–13.2 µg/kg, respectively [14]. A study in China examined 117 samples of edible and medicinal plant seeds and reported that the AFB1 levels detected in Coicis Semen, Hordei Fructus Germinatus, Ziziphi Spinosae Semen, and Semen Perisicae were 6.2, 0.2, 10.3, and 0.3 µg/kg, respectively [15]. 

As a result of aflatoxin contamination in medicinal herbs, the European Commission Regulation 165/2010/EC has implemented a maximum residue level (MRL) of 5 µg/kg for AFB1 and 10 µg/kg for total aflatoxins in some botanical species [16], and the same limits have been set by China [17]. In India, the maximum level of total aflatoxins was set at 30 µg/kg for spices [18]. However, in some countries, no specific MRL for aflatoxins in medicinal herbs has been established. For example, a MRL of 20 µg/kg for the sum of aflatoxins has been set by the USA for all foodstuffs intended for human consumption, except milk [19], and by Thailand for all foods other than peanuts [20]. To ensure the food safety of and to satisfy the consumer requirements for medicinal herbs, a simple and cost-effective analytical method is needed for routine analysis and monitoring of aflatoxins. 

Dispersive liquid–liquid microextraction (DLLME) is a main category of liquid-phase microextraction, and is efficient and environmentally friendly due to its low solvent consumption [21]. DLLME is generally based on the ternary solvent system, in which a small volume of extraction and disperser solvents is rapidly injected into an aqueous sample containing the target compound. Then, a cloudy solution is formed, and the analyte in the sample is extracted into the fine droplets of the extraction solvent. After centrifugation, the enriched analyte in the organic layer is determined based on suitable analytical techniques [22]. DLLME is a recent method that has been used for the extraction and pre-concentration of different mycotoxins in various food matrices, with subsequent quantification based on HPLC-FLD [23,24,25].

To date, there has been a lack of reporting on extraction and pre-concentration based on the DLLME procedure for the quantification of aflatoxins in medicinal herbs using HPLC-FLD. Therefore, the objective of this study was to develop a simple, reliable, and effective DLLME for aflatoxins in dried senna leaves and pods before their determination, based on HPLC-FLD, with the pre-column derivatization method using trifluoroacetic acid (TFA). Aflatoxins in the sample were directly extracted using acidified aqueous methanol. The resulting supernatant was subjected to the DLLME process for the purification and enrichment of aflatoxins. This work optimized the effect of various parameters of the proposed DLLME method. Finally, the optimized method was validated and applied to the determination of aflatoxins in commercially dried senna leaves and pods. As far as we know, this is the first study on the DLLME process of aflatoxins in senna leaves and pods. Moreover, distilled water was used for the first time as a dispersive solvent in the DLLME process.

## 2. Results and Discussion

Several factors may affect the extraction efficiency of the DLLME process, including the type of extraction and dispersive solvents, the addition of salt for the salting-out process, and the pH of the aqueous solution. The extraction solvent is an important factor affecting the DLLME efficiency [26]. In the present study, chloroform was selected as an extraction solvent for DLLME due to its high capability to extract aflatoxins [27,28]. The effects of DLLME parameters were studied and optimized to obtain the most effective extraction. The enrichment factor (EF) and extraction recovery (ER) were used to evaluate the efficiency of the optimization and validation, respectively, of DLLME.

### 2.1. Selection of Dispersive Solvent

**Figure 1:**
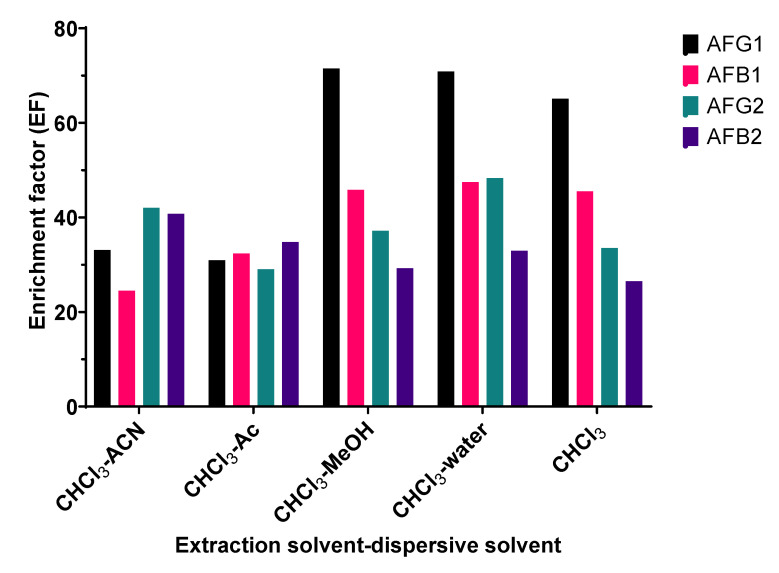
Effect of types of dispersive solvent on EF values of aflatoxins. CHCl_3_: chloroform; ACN: acetonitrile; Ac: acetone, and MeOH: methanol.

The selection of appropriate dispersive solvents was important for performing the DLLME process. In traditional DLLME, the dispersive solvent must be water-miscible and polar solvent, and should be immiscible with an extraction solvent [29]. Acetonitrile, acetone, and methanol are generally used as dispersive solvents. A small volume of the extraction solvent is dispersed into fine droplets in the aqueous phase to form a cloudy solution, assisted by a disperser [30]. In this work, acetonitrile, acetone, methanol, and distilled water were investigated as dispersive solvents and compared to a group with no dispersant. The EF values of aflatoxins with different dispersive solvents are shown in Figure 1. We found that using distilled water as a dispersant provided the highest EF in most aflatoxins, followed by using methanol and no dispersant, respectively. In the case of acetonitrile and acetone, the EF values of some aflatoxin derivatives did not increase compared to the no-dispersant treatment. Although water is not mutually soluble with chloroform, it can also increase the large surface area between fine droplets of chloroform and the water sample. These observations might have been due to the presence of a dispersive solvent decreasing the polarity of the aqueous sample [31], which consequently decreased the transfer of aflatoxins from the aqueous solution into small droplets of chloroform. Therefore, distilled water was chosen as the dispersive solvent in the present study.

### 2.2. Optimization of DLLME Parameters

The results of the independent variables (the volume of chloroform, the salt addition, and the pH of the aqueous sample) and their EF responses for all tested aflatoxins are shown in Table 1. The EF values of AFG1, AFB1, AFG2, and AFB2 were in the ranges of 16.88–132.98, 36.08–139.73, 21.71–96.26, and 49.66–142.77, respectively. The experimental EF values were subjected to analysis of variance (ANOVA), and then multiple linear regression models were developed. 

The second-order polynomial equations that described the EF values of each aflatoxin as a function of the volume of chloroform (X_1_), salt addition (X_2_), and the pH of the aqueous solution (X_3_) were:

EF_AFG1_ = 81.27 − 24.11X_1_ − 18.66X_2_ +5.33X_3_ +15.11X_1_^2^ − 15.06X_2_^2^ − 21.04X_3_^2^ +11.08X_1_X_2_ − 1.80X_1_X_3_ +2.32X_2_X_3_

EF_AFB1_ = 71.61 − 29.39X_1_ − 17.15X_2_ +3.29X_3_ +13.61X_1_^2^ − 9.35X_2_^2^ − 11.18X_3_^2^ +17.52X_1_X_2_ − 0.81X_1_X_3_ +0.33X_2_X_3_

EF_AFG2_ = 72.03 − 17.36X_1_ − 11.66X_2_ +2.1X_3_ +11.5X_1_^2^ − 16.53X_2_^2^ − 21.81X_3_^2^ +1.5X_1_X_2_ +1.13X_1_X_3_ − 3.87X_2_X_3_

EF_AFB2_ = 97.76 − 28.05X_1_ − 10.53X_2_ +4.1X_3_ +11.67X_1_^2^ − 14.08X_2_^2^ − 20.62X_3_^2^ +11.83X_1_X_2_ − 2.66X_1_X_3_ − 2.1X_2_X_3_

As shown in Table 2, the *p*-values less than 0.05 and the high F-values for the models of all aflatoxins indicated that the results of the developed models were significant. In addition, the lack-of-fit values were not significant (*p*-value > 0.05), indicating that the model was adequate for predicting the responses. Goodness-of-fit of the proposed models was also evaluated based on *R*^2^, adjusted *R*^2^, and predicted *R*^2^ [32], which were all greater than 0.90 for all aflatoxins. A suitable model should have an *R*^2^ no less than 0.75 [33]. The high *R*^2^ values demonstrated the high reliability of the model in predicting the ER during the DLLME process. The similarly high values for adjusted *R*^2^ confirmed good agreement between the experimental and predicted values. The differences between the adjusted and predicted *R*^2^ values for each model were less than 20%, indicating that the adjusted *R*^2^ values were in reasonable agreement with the predicted *R*^2^ values [34].

Furthermore, the results of the ANOVA indicated the significance of the linear, quadratic, and interaction model terms. A low *p*-value indicates that the combined effects of all independent variables contributed significantly to predicting the response. It was observed that the linear terms of volume of chloroform (X_1_), salt addition (X_2_), pH of the aqueous solution (X_3_), and their quadratic terms (X_1_^2^, X_2_^2^, and X_3_^2^), as well as the interaction term between the volume of chloroform and salt addition (X_1_X_2_), were significant (*p*-values < 0.05) and affected the EF values of AFG1 and AFB2. For EF values of AFB1 and AFG2, except for X_3_ and its interaction effect with the volume of chloroform (X_1_X_3_) and salt addition (X_2_X_3_), the *p*-values of all linear, quadratic, and interaction terms were significant (*p*-value < 0.05). 

The 3D response surface plots were constructed to describe the effect of the combination of the volume of chloroform, salt addition, and pH of aqueous solution on the EF values of all aflatoxins (Figure S1). Each response surface plot demonstrated the effect of two independent variables on the EF values, while the other variable was fixed at the zero level.

The volume of the extraction solvent has a significant impact on extraction recovery in DLLME process [35]. In the present study, we found that increasing the volume of the extraction solvent did not improve the extraction efficiency. The highest EF of all aflatoxins was obtained when the chloroform volume was 200 µL; then, the EF decreased with an increase in the volume of chloroform. These results were in agreement with previous reports [27,36]. The decrease in EF might have been related to the decrease in the number of fine droplets available for extraction.

The salt concentration is a factor with an impact on the performance of the DLLME; we used NaCl, as it is the most commonly used salt. Generally, salt addition can correspondingly increase or decrease the EF of analytes by salting-out or salting-in [37]. In the present study, no addition of salt improved the result, as higher salinity resulted in a stronger salting-out effect while concurrently reducing the solubility of the extraction solvent, which would have a negative impact on the dispersal of the extraction solvent [38].

The pH of an aqueous solution plays a role in the solubility and ionization of target aflatoxins, resulting in a change in the distribution ratio of the analytes between the organic phase and the aqueous phase [39]. We found that the mean EF of all aflatoxins first increased by increasing the pH from 3 to 5, and then gradually decreased when the pH was greater than 5. This was consistent with a previous study [28] which used vortex-assisted dispersive liquid–liquid microextraction and found that the recoveries of mycotoxins increased gradually with the increase in pH, reaching maximum values at around pH 5, after which they started to decrease.

**Figure 2:**
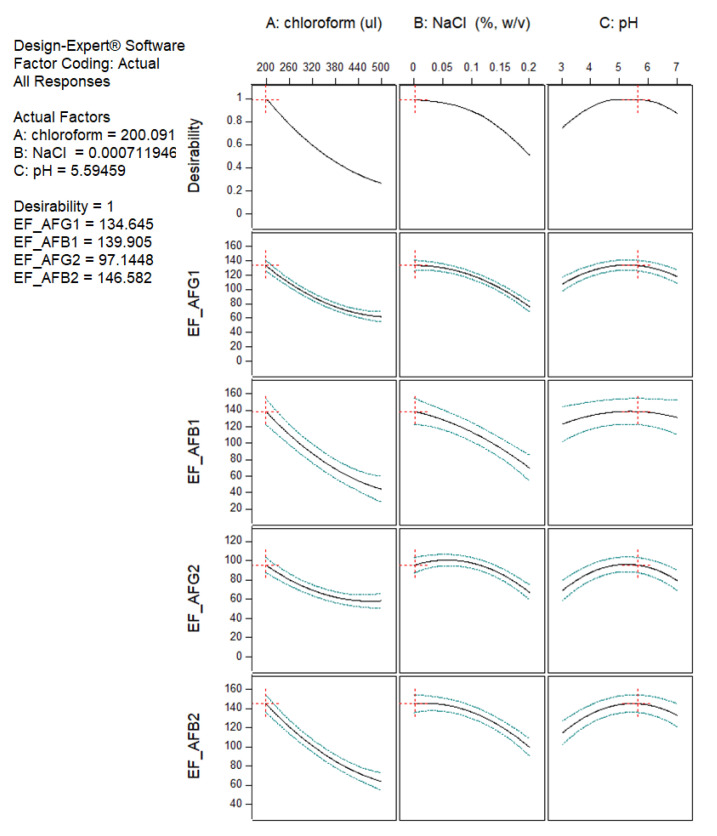
Predicted values and desirability function for maximum EF values of aflatoxins.

The process of determining the optimum DLLME condition based on the highest EF value of all aflatoxins was performed with numerical optimization using the Design Expert software. The results of the desirability analysis showed that a desirability value of 1 could be achieved from 45 solutions. The optimum pH of an aqueous solution obtained from the desirability analysis was predicted within the range 4.91–5.75; however, a pH value of 5.6 was selected due to the distilled water typically used in the laboratory having a pH of approximately 5.6. Therefore, the selected optimum conditions consisted of a chloroform volume of 200 µL, no salt addition, and a pH of the aqueous solution of 5.6. The predicted EF and desirability values of the aflatoxins are shown in Figure 2.

### 2.3. Method Validation

#### 2.3.1. Matrix Effect (ME)

The determination of mycotoxins in dried medicinal herbs involves specific problems, because the herbs contain various pigments as well as many constituents [40]. The undetected compounds in the sample can affect the measured concentration of the target analytes. Similarly to various herbs, dried senna has a high content of natural organic compounds. This matrix can co-extract during the extraction process and substantially disrupt the chromatographic analysis [41]. In this study, ME was assessed using the post-extraction spike method by comparing the slopes of matrix-matched calibration curves with the slopes of calibration curves obtained from standard solutions. As shown in Table 3, all target aflatoxins had negative ME values, indicating that the detector responses for the analysts were decreased or suppressed. Medium negative ME values (between −50% to −20%) were observed in the senna leaf matrix for all aflatoxins in the range from −33.70% to −23.78%. In the senna pod matrix, AFB1 had a medium ME value of −22.65%, while the other aflatoxins had weak ME values (between −20% to 20%) in the range from −18.23% to −17.29%. Similar negative ME values have been reported during the analysis of aflatoxins in different medicinal herb matrices using liquid extraction, followed by C18 cleanup [42], QuEChERs extraction, and dispersive solid phase (dSPE) cleanup [43,44]. In the present study, although some aflatoxins had ME values within ±20%, which is considered not significant [45], the matrix-matched calibrations were applied to achieve accurate and precise quantification.

#### 2.3.2. Linearity, Sensitivity, Accuracy, and Precision

**Figure 3:**
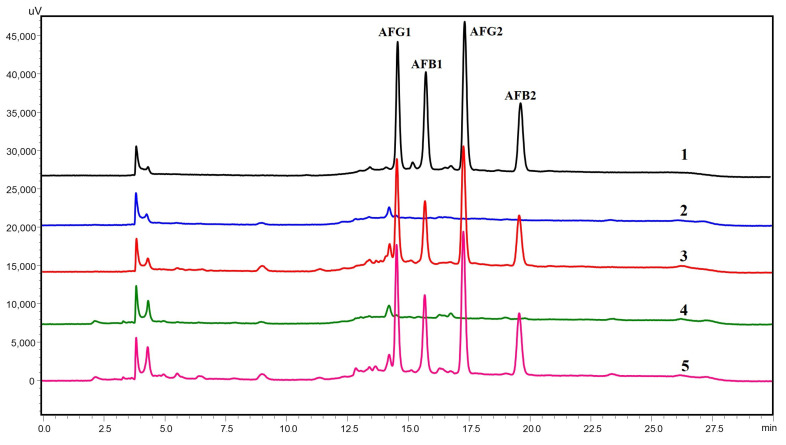
HPLC chromatograms of standard solution containing 2 ng/mL for each aflatoxin (1), blank senna leaves (2), spiked senna leaves (3), blank senna pods (4), and spiked senna pods (5). The spiked concentration for each aflatoxin was 10 µg/kg, which corresponded to a final concentration of 2 ng/mL for each aflatoxin in the assay.

The matrix-matched calibration curves were also used to determine the linearity of the method. A good linear relationship between peak area and concentration was found in the range of 2–50 µg/kg for all aflatoxins in both senna matrices, as shown in Table 3. The *R*^2^ value obtained was greater than 0.995, which indicated good linearity of the developed method. In addition, this linear range covered the regulation limits of aflatoxins in medicinal herbs. The sensitivity of the method was evaluated by calculating the LOD and LOQ for each aflatoxin. The LOD and LOQ values for all aflatoxins in the senna leaves and pods were in the ranges of 0.70–1.27 µg/kg and 2.13–3.84 µg/kg, respectively, which were below the most commonly set limits for AFB1 of 5 µg/kg and total aflatoxins of 10 µg/kg [17]. The accuracy and precision of the optimized method were determined by spiking blank senna leaves and pods at concentrations of 5, 10, and 20 µg/kg of each aflatoxin. As shown in Table 4, the satisfactory recoveries for all aflatoxins in the two senna matrices were in the range of 95.23–105.33%. Intra- and inter-day precisions, calculated as the RSDs of the spiking experiment, were obtained in the ranges of 1.79–2.26% and 2.07–3.01%, respectively, which indicated good precision of the method. The HPLC chromatograms of a standard solution of mixed aflatoxins, the blank sample, and the spiked sample are illustrated in Figure 3.

### 2.4. Application of Developed Method to Real Senna Samples

To investigate the applicability of the proposed DLLME method, commercial dried senna leaf samples (*n* = 30) and dried senna pod samples (*n* = 30) were analyzed using the validated method. We found that none of the senna leaves or senna pods contained aflatoxins. In Thailand, limited studies have been performed on aflatoxins in medicinal herbs, especially senna leaves and pods. Tassaneeyakul, et al. [46] found herbal samples that were contaminated, with the level of total aflatoxins in the range of 1.7–14.3 µg/kg. Among the 2 analyzed senna pods, AFB1 was detected in 1 sample at 2.2 µg/kg. The possible absence of aflatoxins in the senna samples in Thailand could have been due to the lack of aflatoxigenic *Aspergillus* in the samples [47]. Notably, high levels of aflatoxins in senna pods have been reported in India [48]. This study found that 25% of the senna pods collected from the Gujarat and Tamil Nadu regions contained total aflatoxin levels higher than 10 µg/kg, with a maximum concentration of 255 µg/kg.

## 3. Conclusions

A simple, accurate, and precise method for the analysis of AFB1, AFB2, AFG1, and AFG2 in senna leaves and pods was developed. The DLLME process was optimized for simultaneous extraction and pre-concentration before their determination using HPLC-FLD with pre-column derivatization. The method’s validation parameters were within the acceptable ranges and limits for aflatoxins set by the European Commission, the US Food and Drug Administration, and Thailand, thus confirming the satisfactory performance of the DLLME method for the quantification of such mycotoxins in senna leaves and pods. The developed method could be used in quality control laboratories for routine analysis of aflatoxins in medicinal herbs. In addition, this method was used to monitor aflatoxins in senna leaves and pods marketed in Thailand. None of the senna samples contained a detectable level of aflatoxins. 

## 4. Material and Methods

### 4.1. Chemicals and Reagents

The analytical standards of AFB1, AFB2, AFG1, and AFG2 were purchased from the Trilogy analytical laboratory (Washington, MO, USA). All aflatoxin standards were dissolved, diluted in methanol, and stored at −20 °C until use. Acetonitrile (HPLC grade), methanol (HPLC and AR grades), and acetic acid (AR grade) were obtained from Macron Fine Chemicals (Center Valley, PA, USA). Chloroform (AR grade), acetone (AR grade), and trifluoroacetic acid (TFA) (reagent grade) were purchased from Fisher Scientific (Loughborough, UK). 

### 4.2. Sample Extraction

For the evaluation of accuracy and precision, 2 g of each sample of dried senna leaves and pods was weighed and placed into a 50 mL conical tube. The sample was spiked with a standard solution of mixed aflatoxins to obtain 3 different concentrations of each aflatoxin (5, 10, and 20 µg/kg). Each sample was incubated at room temperature to allow for solvent evaporation. Then, 20 mL of 80% (*v*/*v*) methanol containing 1% (*v*/*v*) acetic acid was added. The sample and solvent were vigorously vortex-mixed for 2 min. The mixture was allowed to settle for 5 min; then, the supernatant was passed through filter paper (Whatman No. 1) and subjected to the DLLME process as described in Section 4.4.

A mixed powder of senna leaves and pods in a 1:1 ratio was used for the optimization of the DLLME process. The sample was extracted as described above. The sample extract was spiked with a mixed standard solution of aflatoxins to obtain a final concentration of 2 ng/mL of each aflatoxin, and then used as a sample for DLLME optimization.

### 4.3. Optimization of DLLME Process

A combination of extraction solvent and dispersive solvent was tested in 500 µL of chloroform with 500 µL of each selected dispersive solvent (acetone, acetonitrile, methanol, distilled water, or no dispersive solvent). The experiment was performed using 6 mL of the aqueous sample, comprising 5 mL of distilled water and 1 mL of the diluted sample extract (1:1 diluted with methanol). After performing the DLLME process, the aflatoxin concentration was analyzed. The enrichment factor (EF) was used to evaluate the extraction efficiency under different DLLME conditions [37], which was calculated as:

Enrichment factor (EF) = C_f_/C_i_ (1)

where C_f_ and C_i_ are the concentration of the analyte in the final organic phase and the initial concentration within the sample, respectively. C_f_ was calculated using the direct calibration curves obtained from the standard solutions.

A 3-level, 3-factor Box–Behnken design based on response surface methodology was used for the optimization of the DLLME parameters: volume of chloroform (X_1_), salt addition (X_2_), and pH of aqueous solution (X_3_). The EF values of each aflatoxin were chosen as response factors. Design Expert software version 10 (Trial version; Stat-Ease Inc.; Minneapolis, MN, USA) was used to analyze the experimental data. In total, 15 experiments were randomly performed, each of which contained 3 repetitions at the center point. The experimental data were used to determine the regression coefficients in order to generate the quadratic polynomial model for the prediction of the optimal condition.

### 4.4. DLLME Process

The sample extract was diluted with methanol (AR grade) at a ratio of 1:1. Then, 1 mL of diluted sample was added into a 15 mL conical tube containing 5 mL of distilled water (pH~5.6). Then, a total of 700 µL of the pre-mixture solution, containing 200 µL of chloroform as an extraction solvent and 500 µL distilled water as a dispersive solvent, was rapidly injected using a Hamilton microsyringe into a 15 mL conical tube containing some of the aqueous sample. The resulting mixture was vortexed for 1 min and then centrifuged at 4500 rpm for 5 min. After that, the upper layer of the aqueous solution was removed using a Pasteur pipette. The sedimented layer phase was collected in a new 1.5 mL microcentrifuge tube. The mixture was evaporated until dry in a water bath at 65 °C under a fume hood. 

### 4.5. Derivatization

The dried residue was derivatized by adding 200 µL of n-hexane and then vortexing the mixture for 30 s. TFA (50 µL) was added, homogenized using vortexing for 30 s, and then incubated for 20 min at 25 °C. After that, 450 µL of distilled water containing 1% (*v*/*v*) acetic acid was added and then mixed thoroughly. Finally, the mixture was centrifuged at 4500 rpm for 5 min, and the lower layer was transferred into a vial for HPLC analysis. The sample was stored at −20 °C until analysis.

### 4.6. HPLC-FLD Analysis

HPLC analysis was carried out using the Shimadzu HPLC system (Shimadzu Corp.; Kyoto, Japan), consisting of quaternary pumps (10ATVP), an online degasser (DGU-14A), an auto-injector (SIL-10ADVP), a column oven (CTO-10ASvp), a fluorescence detector (RF-10AXL), and the LC Solution software (Shimadzu, Kyoto, Japan). The excitation and emission wavelengths were 360 and 450 nm, respectively. Separation was performed on an Inertsil ODS-3 C18 Column (250 mm × 4.6 mm, 5 µm) (GL Sciences; Tokyo, Japan) equipped with Ascentis^®^ C18 Supelguard^™^ (2 cm × 4 mm, 5 µm) (Supleco; Bellefonte, PA, USA). The column temperature was controlled at 35 °C. The injection volume was 20 µL. The chromatographic separation was performed using a mobile phase with a water-to-methanol-to-acetonitrile ration of 6:3:2 (*v*/*v*/*v*), containing 2% (*v*/*v*) acetic acid as solvent A and 1% (*v*/*v*) acetic acid in water as solvent B, at a flow rate of 1 mL/min. The gradient elution program was: 0 to 5 min 45%A; 5 to 7 min, 45 to 100%A; 7 to 20 min 100%A; 20 to 22 min, 100 to 45%A; and 22 to 30 min, 45%A. 

### 4.7. Method Validation

The matrix effect (ME) was assessed by comparing the slopes of the calibration curves for the aflatoxin standards prepared in solvent against the standards prepared in sample extract of blank senna leaves and pods, and final concentrations were determined to fall in the range of 2–50 µg/kg. ME was expressed as signal suppression or enhancement using the following equation:

ME (%) = [(X_1_ − X_2_)/X_2_] × 100 (2)

where X_1_ and X_2_ are the slopes of the calibration curves of each aflatoxin prepared in matrix extract and solvent, respectively.

Validation of the present method was performed according to the European Commission Regulation (EC) No 401/2006 [49]. The proposed DLLME process was validated in-house for linearity, limit of detection (LOD), limit of quantification (LOQ), recovery, and precision. Linearity was evaluated by preparing matrix-matched calibration curves in the sample extracts of blank senna leaves and pods, with 5 different concentrations of 0.2, 0.5, 1, 2, and 5 ng/mL and correspondence to 2, 5, 10, 20, and 50 µg/kg of each aflatoxin in the sample matrix. Each curve was prepared and injected in three replicate experiments. The curves were constructed by plotting the peak area of each aflatoxin against the concentrations. The coefficient of determination (*R*^2^) was considered appropriate when *R*^2^ > 0.99. The LOD and LOQ were estimated based on the standard deviation of the y-intercept and the slope of the matrix-matched calibration curves according to the following equations:

LOD = 3.3 × (σ/S)(3)

LOQ = 10 × (σ/S)(4)

where σ is the standard deviation of the y-intercept and S is the slope of a matrix-matched calibration curve.

The accuracy of the method was determined by analysis of each spiked sample, with 6 replicates. The acceptance recovery values of the spiked samples for concentrations of 1–10 µg/kg and >10 µg/kg were set to be within the ranges of 70–110% and 80–110%, respectively. Recovery values were calculated based on a comparison between the obtained concentration and the added amount, using the following equation:

Recovery (%) = [C _spiked sample_/C _expected_] × 100 (5)

where C _spiked sample_ is the mean concentration of the spiked sample calculated using the matrix-matched calibration curve, and C _expected_ is the known value of the added concentration.

Intra-day precision was analyzed with a recovery of spiked senna leaves and pods of 3 different concentrations, using 6 replicates, on the same day. Inter-day precision was performed by repeating the intra-day precision for 3 days with 2 samples per day. The results were calculated as relative standard deviations (%RSD). An RSD value of less than 20% was acceptable for intra-day and intra-day precision [50].

## Figures and Tables

**Table 1:** Box–Behnken experimental design for evaluating the effect of DLLME parameters on the enrichment factors (EFs) of aflatoxins.

Run	Independent Variables			Responses: Enrichment Factor (EF)			
	X_1_: Chloroform Volume (µL)	X_2_: NaCl (%, *w*/*v*)	X_3_: pH	AFG1	AFB1	AFG2	AFB2
1	−1 (200)	−1 (0)	0 (5)	132.98	139.73	96.26	142.77
2	1 (500)	−1 (0)	0 (5)	62.67	43.13	57.44	66.58
3	−1 (200)	1 (0.2)	0 (5)	77.78	73.56	73.56	100.46
4	1 (500)	1 (0.2)	0 (5)	51.79	47.06	40.74	71.59
5	−1 (200)	0 (0.1)	−1 (3)	92.29	98.06	77.82	111.55
6	1 (500)	0 (0.1)	−1 (3)	47.59	43.66	41.93	57.18
7	−1 (200)	0 (0.1)	1 (7)	106.68	106.03	79.23	125.78
8	1 (500)	0 (0.1)	1 (7)	54.77	48.41	47.87	60.76
9	0 (350)	−1 (0)	−1 (3)	62.92	66.74	40.92	68.95
10	0 (350)	1 (0.2)	−1 (3)	16.88	28.60	21.71	49.66
11	0 (350)	−1 (0)	1 (7)	68.82	72.88	53.41	80.66
12	0 (350)	1 (0.2)	1 (7)	32.08	36.08	18.71	52.97
13	0 (350)	0 (0.1)	0 (5)	84.12	83.30	74.99	103.06
14	0 (350)	0 (0.1)	0 (5)	78.41	66.73	67.50	94.29
15	0 (350)	0 (0.1)	0 (5)	81.28	64.79	73.60	95.94

**Table 2:** ANOVA results for Box–Behnken design.

Source	df	AFG1		AFB1		AFG2		AFB2	
		F-Value	*p*-Value	F-Value	*p*-Value	F-Value	*p*-Value	F-Value	*p*-Value
Model	9	126.39	0.000 *	27.83	0.001 *	63.15	0.000 *	73.54	0.000 *
X_1_	1	454.35	0.000 *	142.34	0.000 *	197.41	0.000 *	385.06	0.000 *
X_2_	1	270.55	0.000 *	48.45	0.001 *	89.09	0.000 *	54.29	0.001 *
X_3_	1	22.21	0.005 *	1.79	0.239	2.90	0.149	8.24	0.035 *
X_1_^2^	1	82.20	0.000 *	14.09	0.013 *	39.95	0.001 *	12.35	0.009 *
X_2_^2^	1	81.79	0.000 *	6.65	0.049 *	82.56	0.000 *	44.79	0.001 *
X_3_^2^	1	159.56	0.000 *	9.51	0.027 *	143.86	0.000 *	96.00	0.000 *
X_1_X_2_	1	47.95	0.001 *	25.30	0.004 *	0.74	0.430	34.24	0.002 *
X_1_X_3_	1	1.27	0.311	0.05	0.826	0.42	0.546	1.74	0.245
X_2_X_3_	1	2.11	0.206	0.01	0.928	4.91	0.078	1.08	0.347
Error	5								
Lack-of-Fit	3	1.43	0.437	0.11	0.944	0.61	0.668	0.59	0.679
Pure error	2	-	-	-	-	-	-	-	-
*R* ^2^		0.995		0.980		0.991		0.992	
Adjusted *R*^2^		0.987		0.945		0.975		0.979	
Predicted *R*^2^		0.949		0.916		0.923		0.935	

* *p*-value ≤ 0.05.

**Table 3:** Matrix effect, linear range, LOD, and LOQ of proposed DLLME for aflatoxins in dried senna leaves and pods.

	Matrix Effect (%)	Linear Range (µg/Kg)	Matrix-Matched Calibration Curve	*R* ^2^	LOD (µg/kg)	LOQ (µg/kg)
Dried senna leaves						
AFG1	−23.93	2–50	y = 14,070x + 4958	0.999	1.22	3.70
AFB1	−33.70	2–50	y = 8976x + 434	0.999	1.27	3.84
AFG2	−23.78	2–50	y = 19,187x + 0.39	0.997	0.70	2.13
AFB2	−30.28	2–50	y = 10,265x − 639	0.998	1.02	3.09
Dried senna pods						
AFG1	−17.01	2–50	y = 12,186x + 3553	0.999	1.03	3.14
AFB1	−22.65	2–50	y = 7716x − 208	0.998	0.86	2.60
AFG2	−17.29	2–50	y = 18,252x − 1245	0.996	1.13	3.42
AFB2	−18.23	2–50	y = 8537x + 226	0.997	1.14	3.44

**Table 4:** Accuracy and precision of the proposed DLLME for aflatoxins in dried senna leaves and pods.

	Spiked Level (µg/kg)	Dried Senna Leaves			Dried Senna Pods		
		Recovery (%) (*n* = 6)	RSD (%)		Recovery (%) (*n* = 6)	RSD (%)	
			Inter-Day (*n* = 6)	Intra-Day (*n* = 6)		Inter-Day (*n* = 6)	Intra-Day (*n* = 6)
AFG1	5	103.98	3.23	4.57	99.36	5.55	7.85
	10	92.43	4.67	4.92	96.89	6.53	9.23
	20	91.77	3.41	4.47	91.91	7.18	10.15
AFB1	5	98.47	2.30	3.13	92.18	2.90	4.20
	10	105.06	2.78	3.94	83.50	7.08	10.08
	20	99.22	7.93	10.59	93.99	7.12	9.75
AFG2	5	107.92	2.67	3.91	101.56	5.21	7.27
	10	101.41	5.29	7.49	90.5	6.12	8.91
	20	80.15	7.02	9.15	88.23	3.38	4.39
AFB2	5	103.19	4.41	6.23	102.73	6.93	9.68
	10	108.71	5.21	7.70	94.20	2.47	3.36
	20	92.02	6.58	9.52	89.59	4.92	7.16

## Data Availability

The data that support the findings of this study are available from the corresponding author upon reasonable request.

## Supplementary Materials

The following supporting information can be downloaded at: https://www.mdpi.com/article/10.3390/toxins15040277/s1, Figure S1: Response surface plots presenting the effect of independent variables on EF values of AFG1 (A), AFB1 (B), AFG2 (C), and AFB2 (D): volume of chloroform and salt addition (1), volume of chloroform and pH of aqueous solution (2), and salt addition and pH of aqueous solution (3).
