# Supplementary material for: Optimization and Validation of Dispersive Liquid–Liquid Microextraction for Simultaneous Determination of Aflatoxins B1, B2, G1, and G2 in Senna Leaves and Pods Using HPLC-FLD with Pre-Column Derivatization
Source: Toxins (Basel). 2023 Apr 7;15(4):277. doi: 10.3390/toxins15040277 (PMC10142810; doi:10.3390/toxins15040277)
Supplement: Supplementary file 1 [file toxins-15-00277-s001.zip › toxins-2307203-supplementary.pdf]

Supplementary Materials: Optimization and Validation of Dispersive Liquid–Liquid Microextraction for Simultaneous Determination of Aflatoxins B1, B2, G1, and G2 in Senna Leaves and Pods using HPLC-FLD with Pre-Column Derivatization

Thanapoom Maneeboon, Chananya Chuaysrinule and Warapa Mahakarnchanakul

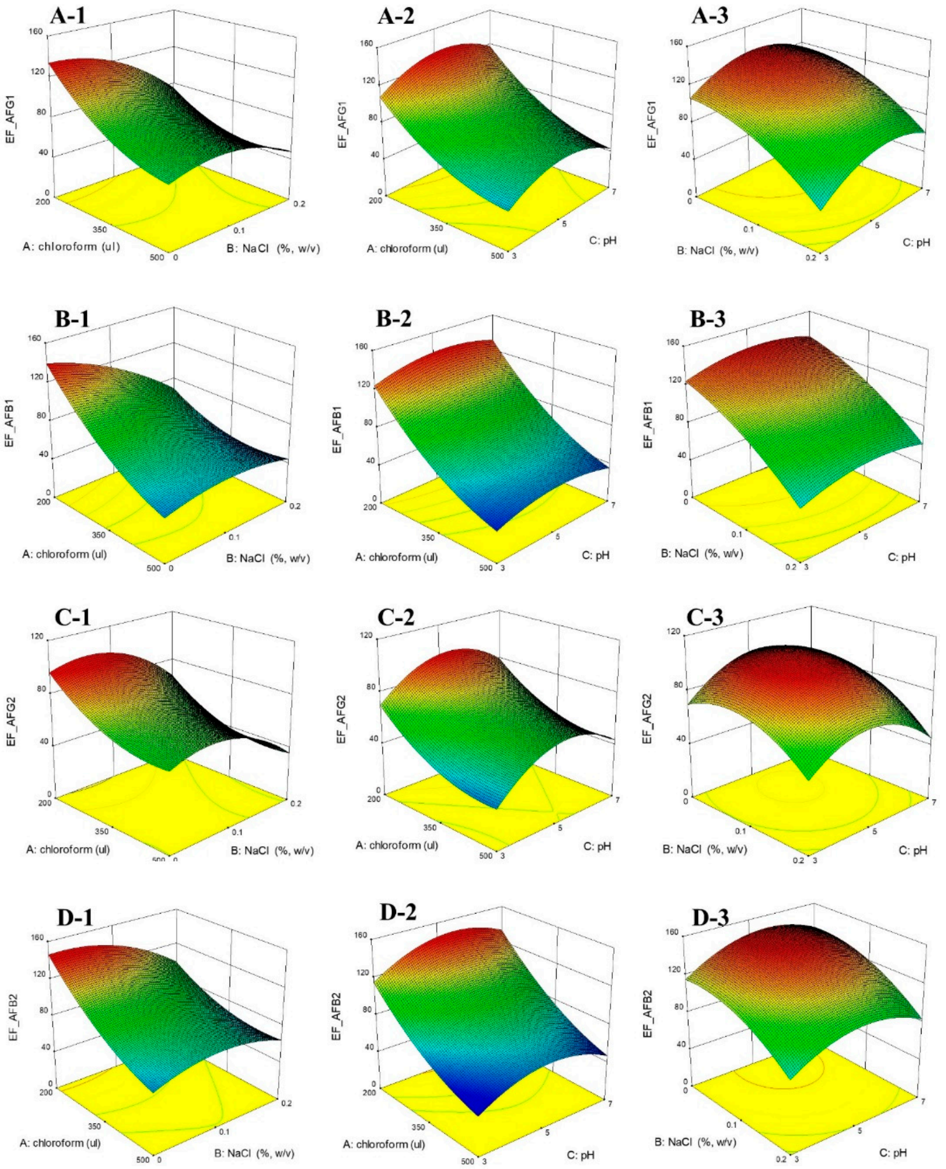

Figure S1. Response surface plots presenting the effect of independent variables on EF values of AFG1 (A), AFB1 (B), AFG2 (C), and AFB2 (D): volume of chloroform and salt addition (1), volume of chloroform and pH of aqueous solution (2), and salt addition and pH of aqueous solution (3)
